# Liver injury in sepsis: manifestations, mechanisms and emerging therapeutic strategies

**DOI:** 10.3389/fimmu.2025.1575554

**Published:** 2025-03-28

**Authors:** Xinqi Xu, Tingyu Yang, Jiapan An, Bin Li, Zhimin Dou

**Affiliations:** ^1^ The First Hospital of Lanzhou University & The First School of Clinical Medicine, Lanzhou University, Lanzhou, China; ^2^ Department of Critical Care Medicine, The First Hospital of Lanzhou University, Lanzhou, China

**Keywords:** sepsis, liver injury, inflammation, oxidative stress, pyroptosis, autophagy, noncoding regulation

## Abstract

Sepsis is defined as a condition related to infection that manifests with multiorgan dysfunction, representing a life-threatening state. Consequently, severe complications frequently occur, with liver injury being one of the most prevalent serious complications of sepsis. Liver dysfunction during sepsis serves as an independent predictor of mortality. This review provides a comprehensive overview of current research on sepsis-induced liver injury (SILI), encompassing the clinical manifestations, diagnostic criteria, pathogenesis and therapeutic strategies associated with this condition. SILI may manifest as hypoxic hepatitis due to ischemia and shock, cholestasis resulting from abnormal bile metabolism, or bile duct sclerosis. The pathophysiology of sepsis involves intricate interactions among the inflammatory response, oxidative stress, and cell death. All of these factors complicate treatment and represent potential targets for therapeutic intervention. Furthermore, this review addresses the limitations inherent in conventional therapies currently employed for managing SILI and emphasizes the potential of novel targeted strategies aimed at addressing the fundamental mechanisms underlying this condition.

## 1 Introduction

Sepsis is a potentially fatal syndrome that arises from multiorgan dysfunction stemming from the host’s dysregulated response to infection. Currently, it has emerged as one of the leading contributors to infection-related mortality worldwide (1). According to epidemiological data, the annual incidence of adult sepsis is approximately 189 cases/100,000 individuals, with a mortality rate as high as 26.7% (2). Notably, 24.4% of sepsis cases occur within the intensive care unit (ICU) (3). Sepsis represents a central focus of medical research because of its high incidence, complex pathogenesis, critical nature, and unfavorable prognosis.

As a crucial metabolic immune organ, the liver clears bacteria and synthesizes acute phase proteins and cytokines during sepsis. Thus, immune defense can be effectively activated through inflammatory responses (4). However, an excessive inflammatory response may cause significant hepatocyte injury. This condition is specifically known as SILI (5). Research has shown that in patients with sepsis complicated by liver dysfunction or failure, the mortality rates range from 54% to 68% (6). These rates are significantly higher than those in patients with respiratory dysfunction or failure (7). Consequently, minimizing liver damage and promoting the recovery of liver function are essential to reduce the mortality rate among patients with sepsis.

With respect to the pivotal role of the liver in the pathophysiology of sepsis, this paper discusses the various types and clinical features of hepatic injury associated with sepsis. It subsequently reviews the critical mechanisms underlying the pathogenesis of hepatic injury, including interactions among inflammatory responses, oxidative stress, mitochondrial dysfunction, pyroptosis, and autophagy. This analysis aims to provide new insights and strategies for addressing hepatic injury in sepsis. More specifically, this paper evaluates current clinical trials and emerging therapies that are grounded in these pathogenic mechanisms. This study provides a theoretical framework and practical reference for future treatment strategies targeting SILI.

## 2 Types of hepatic injury in sepsis and clinical diagnostic criteria

**Figure 1:**
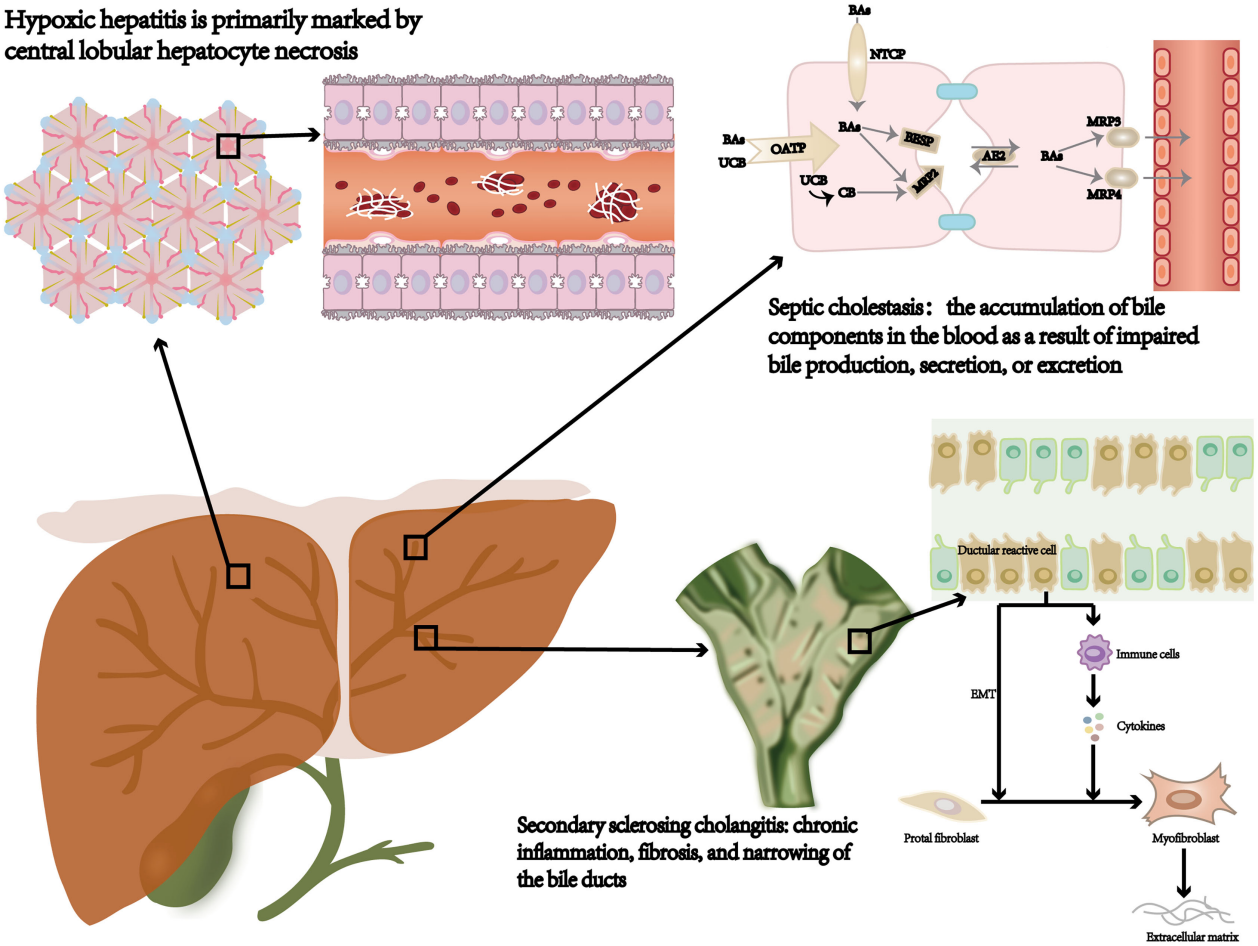
Septic liver injury type: hypoxic hepatitis, septic cholestasis, and secondary sclerosing cholangitis. NTCP, sodium‐taurocholate cotransporting polypeptide; OATP, organic anion transporting polypeptide; AE2, anion exchanger 2; BSEP, bile salt export pump; MDR3/4, multidrug resistance gene 3/4; Bas, bile acids; CB, conjugated bilirubin; UCB, unconjugated bilirubin; EMT, epithelial-mesenchymal transition.

In clinical practice, sepsis may cause various types of liver injury, including hypoxic hepatitis, septic cholestasis, and secondary sclerosing cholangitis (**Figure 1**). These diverse clinical features associated with SILI complicate the diagnostic process. A better understanding of the specific clinical features related to each type is essential for the early and accurate diagnosis of liver injury in sepsis patients.

### 2.1 Hypoxic hepatitis

HH typically develops within 48 hours after the occurrence of heart, circulatory, or respiratory failure. It is distinguished by a marked elevation in aminotransferase levels, frequently exceeding the standard upper limit by more than 10 times while ruling out alternative causes of hepatocellular necrosis (8, 9). Histologically, HH is predominantly characterized by central lobular necrosis of hepatocytes (10). Sepsis-induced systemic inflammation in HH adversely affects cardiac pump function, diminishing blood flow to the liver and consequently reducing the oxygen supply. The most common causes of HH include shock (48%), cardiac arrest (25%), and hypoxia (13%) (8). Laboratory features usually include elevated levels of lactate dehydrogenase (LDH), mildly increased total bilirubin (mainly unconjugated), and a prolonged international normalized ratio (INR), indicating coagulation dysfunction (8, 11, 12).

### 2.2 Septic cholestasis

The SC is defined as the accumulation of bile components in the bloodstream due to impaired bile formation, secretion, or excretion during sepsis (13). Dysfunction is typically related to impaired cholangiocyte function without evidence of significant bile duct damage or hepatocyte loss (14). Laboratory tests revealed that the bilirubin level, mainly direct bilirubin, exceeded 2 μmol/L. Additionally, alkaline phosphatase (ALP) and γ-glutamyltransferase (GGT) levels are elevated to more than 2–3 times the normal range, whereas AST and ALT levels are generally within normal limits (9, 15).

### 2.3 Secondary sclerosing cholangitis

Severe sepsis can lead to SSC, which encompasses chronic inflammation, fibrosis, and stenosis of the bile ducts (16, 17). These pathological changes can ultimately progress to cirrhosis and liver failure (18). More importantly, SSC is positively correlated with severe systemic hypotension, trauma, acute respiratory distress syndrome (ARDS), and systemic inflammatory response syndrome (SIRS) (19, 20). The major clinical features of SSC include chronic cholestasis, thickening of the bile duct walls, and either narrowing or dilation. These manifestations can be diagnosed through abdominal CT scans or magnetic resonance cholangiopancreatography (MRCP) (20–22). A distinction should be made from primary sclerosing cholangitis, which is a chronic autoimmune disease.

Considering the high mortality rate of patients with sepsis who experience complications of liver dysfunction, early identification and prompt intervention are crucial for improving patient prognosis.

## 3 Pathogenesis of hepatic injury in sepsis

The clinical manifestations of hepatic injury in sepsis, such as jaundice, elevated liver enzymes, and prolonged prothrombin time, are closely linked to the underlying pathogenic mechanisms. Understanding these mechanisms can provide insights into why and how liver dysfunction occurs in septic patients. The liver serves a dual function in sepsis: it is responsible for maintaining body homeostasis through the elimination of pathogens and toxins. They are also susceptible to injury due to excessive inflammatory responses and immune dysregulation. The systemic inflammatory cascade in sepsis damages liver cells and impairs their functionality. Moreover, advanced age, alcoholism, diabetes, mellitus and cardiac dysfunction are factors that markedly increase the risk of liver injury in septic patients (23). Therefore, understanding the pathogenesis of SILI is important for elucidating the various clinical manifestations of liver damage observed in sepsis.

**Figure 2:**
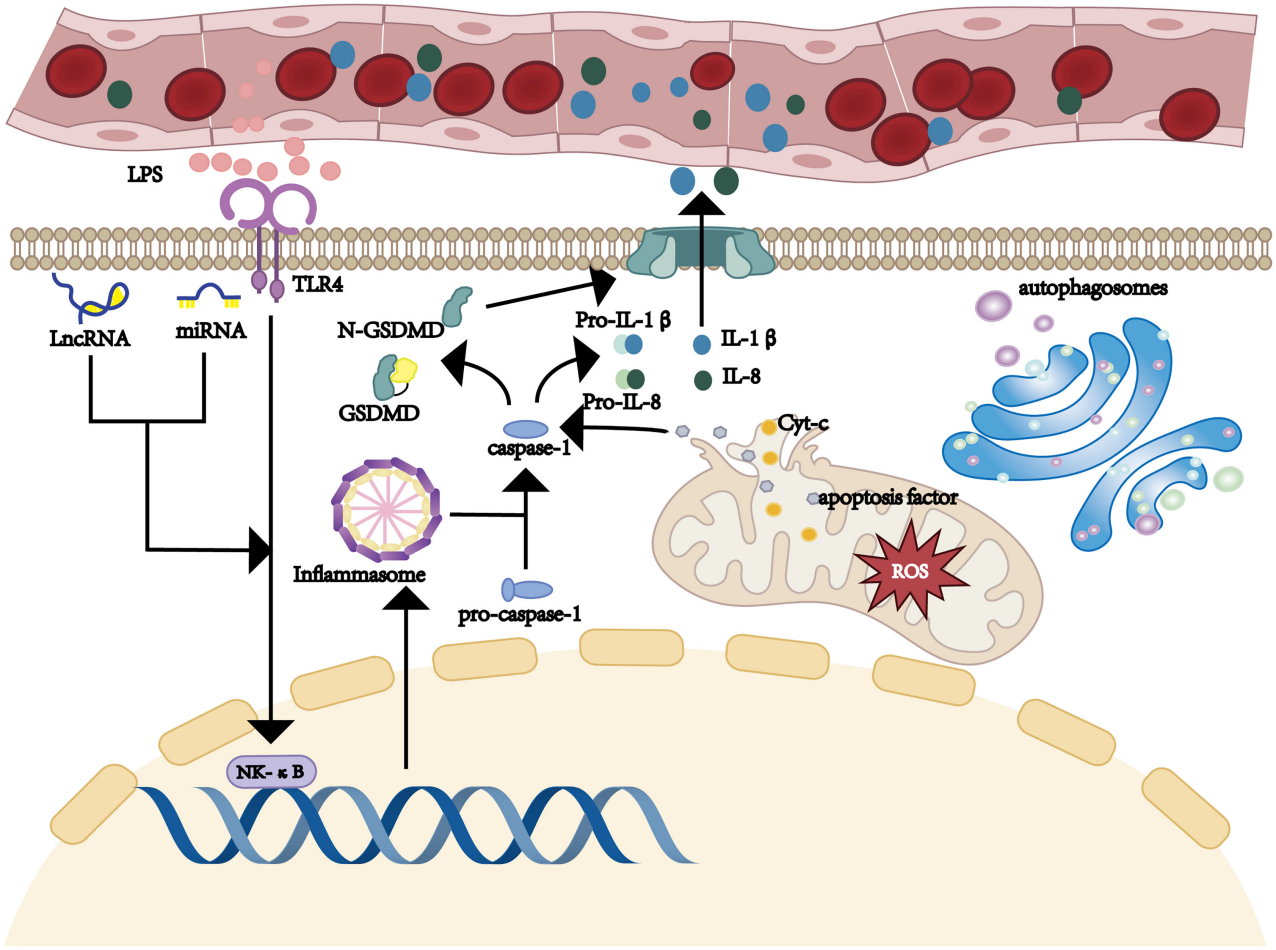
Pathogenesis of hepatic injury in sepsis.

Research has shown that the pathogenesis of SILI is intricately related to inflammation and its subsequent cascade reactions, oxidative stress, pyroptosis, autophagy, and the regulation of microRNAs (miRNAs) (**Figure 2**).

### 3.1 Inflammation and cascade amplification

**Figure 3:**
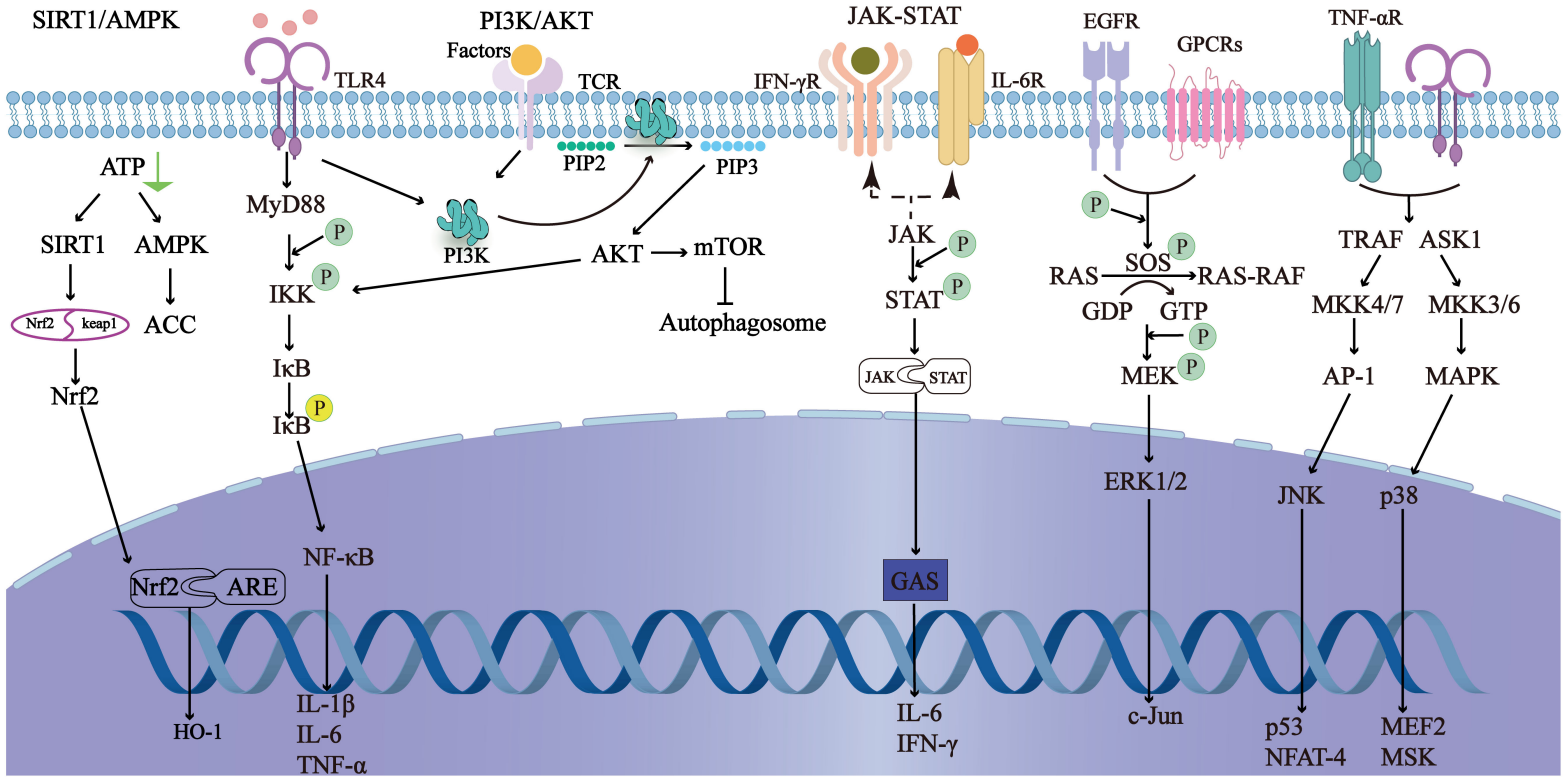
It shows the main related signaling pathways in sepsis-induced liver injury. By regulating the signaling pathways, the inflammatory response can be reduced, autophagy can be initiated, and oxidative stress can be prevented to improve sepsis-induced liver injury.

The inflammatory response and cascade amplification are central aspects of the pathophysiology of SILI (**Figure 3**). Under sepsis conditions, bacteria and viruses produce pathogen-associated molecular patterns (PAMPs), which are recognized by pattern recognition receptors (PRRs) expressed on host cells. Among these receptors are Toll-like receptors (TLRs) (24). Gram-negative bacteria-derived lipopolysaccharides (LPSs) trigger immune cells, including Kupffer cells (KCs) and monocytes/macrophages, via the TLR4-mediated signaling pathway (25–27). This activation enhances the secretion of proinflammatory cytokines, such as tumor necrosis factor-α (TNF-α), interleukin-1β (IL-1β), and interleukin-6 (IL-6). These cytokines not only exacerbate local inflammation but also propagate a systemic inflammatory response, commonly referred to as a “cytokine storm” (28, 29).

Additionally, NOD-like receptors (NLRs) play a crucial role in the inflammatory cascade. NLRs are intracellular PRRs that recognize damage-associated molecular patterns (DAMPs) and PAMPs. Activation of NLRs leads to the formation of inflammasomes, which in turn activate caspase-1 (30). Caspase-1 cleaves pro-IL-1β and pro-IL-18 into their mature forms, further amplifying the inflammatory response. The NF-κB signaling pathway is also a key component of this inflammatory cascade. Upon activation by PAMPs or DAMPs, NF-κB translocates to the nucleus and induces the transcription of proinflammatory cytokines, chemokines, and adhesion molecules, thereby promoting inflammation. Furthermore, infection with gram-positive bacteria, particularly *Staphylococcus aureus*, significantly increased the risk of acute liver injury among septic patients (23, 31).

Cytokines directly induce hepatocyte apoptosis or necrosis (32). Moreover, increased recruitment of immune cells to the inflamed site aggravates the inflammatory response, resulting in a cascade amplification effect that ultimately leads to multiple organ dysfunction syndrome (MODS). As the liver serves as a central metabolic organ, its dysfunction significantly impairs overall bodily function. Thus, targeting the disruption of this amplification cascade may be an effective therapy to alleviate liver injury.

### 3.2 Oxidative stress

Oxidative stress is a pivotal mediator of liver injury in sepsis, exacerbating pathophysiological processes through direct hepatocyte damage and compromising the antioxidant defense system.

In sepsis, the excessive generation of reactive oxygen species (ROS) and reactive nitrogen species (RNS) is a critical factor. Studies have shown that in severe sepsis patients, the levels of ROS can increase by more than 50% compared with those in healthy individuals, while the activities of antioxidant enzymes such as superoxide dismutase (SOD) and catalase (CAT) are significantly reduced, indicating a state of antioxidant depletion (33, 34). ROS interact with proteins, carbohydrates, nucleic acids, and unsaturated lipids in hepatocytes, causing damage. For example, the interaction of ROS with lipids can lead to lipid peroxidation, which disrupts the integrity of cell membranes and organelles. Additionally, ROS can modify proteins, leading to loss of their function and the potential for aggregation, which can further impair cellular processes (35).

The interplay between oxidative stress and inflammation is intricate and forms a vicious cycle. ROS generation exacerbates inflammatory cascades in several ways (36). Firstly, ROS can activate NF-κB, a key transcription factor that induces the expression of pro-inflammatory cytokines such as tumor TNF-α and IL-6 (37). Studies have shown that in sepsis models, the activation of NF-κB can increase the production of TNF-α by more than 2-fold, further amplifying the inflammatory response (38). Secondly, ROS can also promote the activation of NOD-like receptors (NLRs), leading to the formation of inflammasomes and the activation of caspase-1, which in turn cleaves pro-IL-1β and pro-IL-18 into their mature forms, further amplifying inflammation (39).

Furthermore, oxidative stress contributes to endothelial dysfunction, microthrombosis, hepatic sinusoidal obstruction, and diminished hepatic perfusion, which worsen liver injury (40). For example, the increase in ROS can lead to the activation of endothelial cells, promoting the expression of adhesion molecules and the recruitment of leukocytes, which can cause microvascular inflammation and obstruction (41).

A promising strategy may be incorporating antioxidant therapy into the treatment of sepsis to mitigate oxidative stress-induced liver damage. For example, the use of N-acetylcysteine (NAC), a well-known antioxidant, has been shown to reduce ROS levels by more than 40% and increase antioxidant enzyme activities, thereby alleviating liver injury in sepsis models (42).

### 3.3 Mitochondrial dysfunction

Mitochondrial dysfunction represents a critical factor contributing to disturbances in liver energy metabolism and organ damage during sepsis (43). Mitochondrial damage is frequently accompanied by increased oxidative stress, which promotes inflammatory responses and apoptosis. These processes are important mechanisms contributing to the deterioration of both the structure and function of the liver.

LPS, through the activation of hepatic cells, induces an overproduction of ROS, which causes damage to the mitochondrial membrane. This not only interferes with the binding of oxygen by cytochrome oxidase, blocking the respiratory chain and causing metabolic breakdown of liver energy but also triggers the release of cytochrome C, initiating hepatocyte apoptosis (33, 44). In septic mice, the release of cytochrome C from mitochondria can increase by up to 3-fold, significantly promoting apoptosis (44, 45). Liver mitochondria undergo structural injury, including mitochondrial swelling, disorganized crista proliferation, and decreased matrix electron density, in severe sepsis (46). Morphological alterations also include proton leakage, modifications in membrane permeability, and possible disruption of the mitochondrial membrane (33). Among these factors, changes in mitochondrial membrane permeability are critical at the beginning of apoptosis (47). Mitochondria induce changes in membrane permeability by releasing cytochrome C and apoptosis-inducing factors. These releases activate the caspase cascade, leading to the initiation of apoptosis or programmed cell death (48, 49). Regulating mitochondrial function represents a promising therapeutic target for mitigating SILI. This approach may offer a valuable strategy for clinical intervention.

#### 3.3.1 Mitochondrial dynamics

In addition to the structural changes, mitochondrial dynamics, encompassing fusion and fission processes, also play a pivotal role in the progression of SILI. Mitochondrial fusion, which is mediated by proteins such as optic atrophy 1 (OPA1) and mitofusin 2 (MFN2), helps maintain mitochondrial integrity and function by facilitating the exchange of mitochondrial contents and the sharing of mitochondrial DNA (42). During sepsis, the balance between mitochondrial fusion and fission is disrupted, leading to impaired mitochondrial function and increased susceptibility to damage.

Conversely, mitochondrial fission, driven by proteins like dynamin-related protein 1 (DRP1), is essential for the segregation of damaged mitochondria and their subsequent degradation through mitophagy (50). However, excessive fission can result in the fragmentation of mitochondria, reducing their energy production efficiency and increasing the production of ROS. Studies have shown that in septic mice, the expression of DRP1 is upregulated, while the levels of OPA1 and MFN2 are decreased, indicating an imbalance in mitochondrial dynamics. This imbalance contributes to the exacerbation of liver injury by promoting mitochondrial dysfunction and oxidative stress.

Regulating mitochondrial dynamics could be a promising therapeutic strategy for mitigating SILI. For instance, promoting mitochondrial fusion by enhancing the expression of OPA1 or inhibiting excessive fission through the modulation of DRP1 activity may help restore mitochondrial function and reduce liver damage. Further research into the specific mechanisms and potential therapeutic targets related to mitochondrial dynamics in SILI is warranted to provide a more comprehensive understanding of this complex process (51).

### 3.4 Pyroptosis

Pyroptosis is a form of programmed cell death that plays a vital role in the pathogenesis of SILI. The activation of immune cells initiates the pyroptotic pathway, which facilitates the release of inflammatory mediators. In turn, these inflammatory mediators aggravate inflammatory responses and further hepatocyte injury (52).

In the event of an attack by pathogens or inflammatory mediators, hepatocytes release PAMPs or damage-associated molecular patterns (DAMPs). Upon recognition by PRRs, these molecules initiate the activation of inflammasomes, especially the NOD-like receptor protein 3 (NLRP3) inflammasome, promoting the activation of Caspase-1 (53–55). This activation occurs through a two-step process (56). Initially, PRRs detect PAMPs or DAMPs, triggering the nuclear factor-κB (NF-κB) pathway and increasing NLRP3 and pro-IL-1β expression (57). This primes the cell for inflammation. A second signal, such as extracellular ATP or mitochondrial reactive oxygen species (mtROS), subsequently activates the NLRP3 inflammasome (58). Once activated, it recruits and activates Caspase-1 via the ASC adaptor protein, which then processes pro-IL-1β into its mature form for secretion (59). This secretion recruits immune cells to the liver, amplifying inflammation and exacerbating SILI. Activated Caspase-1 not only facilitates the maturation of proinflammatory cytokines such as IL-1β and IL-8 (60–62) but also cleaves gasdermin D (GSDMD), which forms pores in the cell membrane (63). The pores release cellular contents into the extracellular milieu, promoting inflammation and pyroptosis (64–67). The mature IL-1β cytokine recruits innate immune cells to the site of infection and enhances the activation of acquired immune cells. This process amplifies the inflammatory response and exacerbates liver injury. In addition to the classical Caspase-1-mediated pathway, pyroptosis can also be initiated through a nonclassical pathway involving the activation of Caspase-4, Caspase-5, or Caspase-11. Notably, Caspase-4/5 are especially important in humans (55, 68). These caspases can be directly activated by components derived from intracellular pathogens, such as LPS (69), leading to GSDMD cleavage and initiating pyroptosis (54).

Targeting the pyroptosis pathway and preventing its activation offers a promising strategy for reducing liver injury and may provide a novel therapeutic approach for the management of SILI.

### 3.5 Autophagy mechanism

Autophagy is crucial for cellular self-protection and the maintenance of homeostasis by degrading unnecessary proteins and damaged organelles (70). It also plays a vital role in regulating mitochondrial homeostasis by selectively removing damaged mitochondria through mitophagy, which helps maintain cellular energy production and prevent the accumulation of harmful reactive ROS (71, 72).

Mitophagy, the selective autophagy of damaged or dysfunctional mitochondria, is triggered by the loss of mitochondrial membrane potential. This process marks damaged mitochondria with ubiquitin, leading to their recognition and engulfment by autophagosomes. These autophagosomes then fuse with lysosomes, where lysosomal enzymes degrade the contents, ensuring only dysfunctional mitochondria are eliminated while preserving healthy ones for cellular energy needs (73).

Mitophagy is mediated by two main pathways: the ubiquitin-dependent PINK1/Parkin pathway and ubiquitin-independent pathways. In the PINK1/Parkin pathway, PINK1 stabilizes on the outer mitochondrial membrane upon membrane potential loss, recruiting Parkin (74). Parkin ubiquitinates mitochondrial proteins, marking them for degradation (74). Autophagy receptors like p62 recognize these ubiquitinated mitochondria and link them to autophagosomes for clearance (75). Ubiquitin-independent pathways involve mitochondrial proteins such as NIX and BNIP3, which can directly interact with LC3 to initiate autophagosome formation around mitochondria, offering alternative mechanisms for maintaining mitochondrial homeostasis (71, 76).

Research has revealed that SILI is closely associated with impaired autophagy activity in the liver (77). At an early stage of SILI, autophagic flux is increased, resulting in increased accumulation of autophagosomes. However, the number of autophagy-lysosomes decreases, leading to incomplete autophagy and further aggravating liver injury (77). Defective autophagy also results in the accumulation of damaged mitochondria, which further promotes cell death and worsens liver injury (78). When autophagy is overactivated, it may lead to the degradation of essential cellular components, resulting in cell dysfunction and death. In SILI, the excessive production of ROS and the activation of inflammatory signaling pathways can trigger excessive autophagy, which in turn exacerbates liver injury. For instance, the overactivation of autophagy can cause the loss of mitochondrial mass and impair cellular respiration, leading to energy deficiency and cell death (79). These findings suggest that autophagy dysfunction may be critical in the onset and progression of liver injury during sepsis.

Although most research on autophagy remains experimental, its critical role in maintaining cellular homeostasis and alleviating liver injury is undeniable. This pathway can modulate autophagy, particularly in the formation and degradation of autophagosomes, to restore the autophagy-lysosome system. This path offers a novel therapeutic approach for treating liver injury resulting from sepsis. This provides not only a fundamental theoretical basis but also new hope for addressing SILI.

### 3.6 Epigenetic regulation

Noncoding RNAs, including miRNAs and long noncoding RNAs (lncRNAs), modulate mRNA stability and translation efficiency post-transcriptionally by binding to target mRNAs. These RNAs play key regulatory roles in apoptosis, inflammation, and other cellular processes.

Epigenetic factors, such as DNA methylation and histone modifications, also significantly influence hepatic inflammatory responses by altering gene expression patterns without changing the DNA sequence (80). DNA methylation, primarily occurring at CpG islands, can lead to gene silencing by inhibiting the binding of transcription factors or recruiting methyl-binding proteins (81). Aberrant methylation patterns during sepsis affect genes involved in immunity and survival. For example, hypermethylation of the promoter region of the anti-inflammatory cytokine IL-10 reduces its expression, impairing the anti-inflammatory response (82).

Histone modifications, including acetylation, methylation, and phosphorylation, regulate gene transcription by altering chromatin structure (83). Acetylation of histone H3 at the promoter regions of proinflammatory cytokines such as TNF-α and IL-6 enhances their transcription, exacerbating liver injury (84). Conversely, deacetylation by histone deacetylases (HDACs) suppresses inflammatory responses and protects against liver damage (85). These epigenetic changes can create a vicious cycle where inflammation and oxidative stress synergistically worsen liver damage, highlighting the potential of targeting these mechanisms for therapeutic intervention.

Dysregulation of the expression of several miRNAs during sepsis is associated with liver injury. Studies have shown that miR-133a is highly expressed in both septic patients and cecal ligation and perforation (CLP) model mice and that its knockout attenuates liver injury, indicating that miR-133a might play a key role in SILI (86). Increased expression of miR-155 causes the inactivation of nuclear factor E2-related factor 2 (Nrf2), which elevates oxidative stress and exacerbates SILI (87). Additionally, miR-126-5p is highly upregulated in liver cells in both *in vivo* and *in vitro* models of sepsis. The overexpression of miR-126-5p induces cell apoptosis, thereby corroborating its involvement in SILI (88). In contrast, miR-122a has shown sensitivity and specificity in the diagnosis of SILI, suggesting its potential as a biomarker for SILI (89).

Previous studies have preliminarily verified the clinical value of lncRNAs in sepsis (90). For example, the lncRNA CRND is significantly downregulated in septic rats and LPS-treated hepatocytes. This downregulation of the lncRNA CRND might exacerbate the severity of SILI (88). In addition, the lncRNA NEAT1 can compete with Let-7a to regulate TLR4 to promote the development of SILI (91). The expression of the lncRNA MALAT is upregulated in SILI, where it binds to polypyrimidine tract-binding protein 1 (PTBP1). This interaction engages interferon-induced helicase C-domain protein 1 (IFIH1). The binding of these proteins promotes the polarization of M1 macrophages, thereby worsening SILI (92).

These findings illustrate that a complex mechanism underlies the regulatory effects of lncRNAs on SILI. LncRNAs function in SILI by modulating miRNAs through interactions as ceRNAs (also known as miRNA sponges), thereby inhibiting both their expression and function. The lncRNA CRNDE aggravates liver injury by regulating miR-126-5p and BCL2L2 (88). The lncRNA-220-miR-5101-ceRNA complex mediates LPS-induced liver injury through the phosphoinositide 3-kinase (PI3K)/protein kinase B (AKT)/mechanistic target of rapamycin (mTOR) signaling pathway. It influences the regulation of autophagy and apoptosis in Kupffer cells triggered by LPS (93). Similarly, the lncRNA CASC7 facilitates LPS-induced liver injury during sepsis through the miR-217/TLR4 axis (94). Moreover, the lncRNA SNGH11 mediates ferroptosis in SILI via the miR-324-3p/glutathione peroxidase 4 (GPX4) axis (95).

In addition, circRNAs have the capacity to adsorb and neutralize many miRNAs to influence gene expression regulation. For instance, circ-Katnal1 exacerbates inflammatory pyrogenesis in SILI through the miR-31-5p/GSDMD axis (96).

Therefore, dysregulation of the expression of miRNAs and lncRNAs, as well as their interaction, may disrupt critical physiological processes such as the inflammatory response and cell death, contributing to liver injury in sepsis. In this context, a thorough investigation into the roles and mechanisms of these noncoding RNAs is paramount for identifying new potential therapeutic targets.

Notably, all the aforementioned pathogenic mechanisms do not operate in isolation. Rather, they are interconnected and form a complex network of pathogenesis. The close relationship between inflammation and oxidative stress represents a critical element of this network. The inflammatory response triggers the release of numerous inflammatory mediators and cytokines that induce oxidative stress, leading to lipid peroxidation of the cell membrane, protein oxidation, and DNA damage. In turn, oxidative stress further activates inflammatory signaling pathways that enhance the infiltration of inflammatory cells and the secretion of mediators, thus aggravating the inflammatory response. In addition, both inflammation and oxidative stress influence pyroptosis, a form of programmed cell death that is precisely regulated by noncoding RNAs. These mechanisms interact synergistically. Each factor creates a vicious cycle that exacerbates liver damage and SILI. Consequently, a more in-depth investigation into the pathogenesis of SILI is warranted not only to elucidate its intricate pathogenic network but also to provide a scientific foundation for the development of new therapeutic strategies.

In addition to noncoding RNAs, epigenetic modifications such as histone modifications and DNA methylation also significantly influence gene expression and cellular functions during SILI.

Histone modifications, including acetylation, methylation, and phosphorylation, can alter the chromatin structure and accessibility, thereby regulating gene transcription. For example, acetylation of histone H3 at the promoter regions of proinflammatory cytokines such as TNF-α and IL-6 has been shown to enhance their transcription and exacerbate liver injury in sepsis models (97). Conversely, deacetylation of histones by HDACs can suppress inflammatory responses and protect against liver damage (98).

DNA methylation, which primarily occurs at CpG islands, can lead to gene silencing by inhibiting the binding of transcription factors or recruiting methyl-binding proteins. Aberrant DNA methylation patterns have been observed in the liver during sepsis, affecting the expression of genes involved in immune responses and cell survival. Hypermethylation of the promoter region of the anti-inflammatory cytokine IL-10 has been reported in septic patients, resulting in decreased IL-10 expression and impaired anti-inflammatory capacity (99). On the other hand, hypomethylation of the proapoptotic gene Bim has been associated with increased Bim expression and hepatocyte apoptosis in sepsis (100).

These findings highlight the potential of targeting histone modifications and DNA methylation as therapeutic strategies for SILI. For instance, the use of histone deacetylase inhibitors (HDACis) has shown promise in reducing inflammation and improving liver function in sepsis models (101). Similarly, DNA methyltransferase inhibitors (DNMTis) may help restore the expression of beneficial genes and alleviate liver injury (102).

## 4 Treatment of hepatic injury in sepsis

### 4.1 Conventional treatment

The conventional treatment strategy for SILI is systematic and multifaceted to improve outcomes. Central to this therapy is anti-infection treatment. The early initiation of antibiotic therapy is essential for the rapid elimination of pathogens and prevention of infection spread, thereby controlling the progression of SILI (103). Effective management of the infection source by surgical or interventional procedures, such as abscess drainage, can drastically reduce pathogen loads.

Initial fluid resuscitation and hemodynamic support are fundamental components for achieving circulatory stability in SILI patients (104). Nutritional support and metabolic control can improve the intestinal microbiota, enhance the immune response and improve patient recovery (105).

Liver function support constitutes a component of the treatment regimen. This may lead to the incorporation of artificial liver devices that help detoxify and regenerate hepatocytes. Blood purification techniques are employed to eliminate toxins from the body, alleviating the metabolic burden on the liver (106, 107).

However, conventional therapeutic options for SILI present several disadvantages in clinical treatment. With the continuous increase in antibiotic resistance, selecting valid antibiotics has become more complicated, complicating treatment strategies and possibly leading to persistent infections that exacerbate liver damage. Moreover, multiple drugs tend to produce drug interactions that can increase liver and kidney toxicity while limiting available therapeutic options. These factors might further worsen organ dysfunction and negatively impact treatment outcomes.

Moreover, septic liver injury is frequently accompanied by dysfunction in other organs, rendering comprehensive intervention based on single-organ support therapies significantly more challenging. Presently, there is a lack of effective therapeutic targets against the underlying pathophysiological processes involved in SILI. This limitation severely restricts the efficacy of existing treatments and fundamentally hinders improvements in patient prognosis.

### 4.2 Research progress in new therapies

As discussed above, SILI has a complex and multilevel pathogenesis. Effective treatment of SILI can be achieved only by addressing the interconnected pathogenic mechanisms in conjunction with managing liver injury itself. The optimal therapeutic intervention must target various stages of the disease, taking into account not only hepatic and systemic inflammatory responses but also organ dysfunction.

#### 4.2.1 New drug delivery systems

Nanodrug delivery systems utilize nanotechnology to achieve targeted and precise drug delivery to damaged liver tissues, which increases drug bioavailability while minimizing toxic side effects. Different delivery platforms, including liposomes, solid lipid nanoparticles, and polymer micelles, have been developed for the treatment of SILI and have shown promising therapeutic effects. Collectively, these findings provide robust support for the potential clinical application of nanotechnology-based therapies in the management of SILI (108–111).

#### 4.2.2 Immunoregulatory therapy

Immunoregulatory therapies are designed to modulate the activities of immune cells to mitigate the inflammatory injury caused by sepsis. This modulation can be achieved through the administration of monoclonal antibodies that neutralize key inflammatory mediators, such as TNF-α and IL-6 (112). Alternatively, IL-1 receptor antagonists may be employed to inhibit the signaling pathways involved in inflammation (113, 114). Furthermore, the Decoy Receptor 3 (DcR3) analog DCR3-SUMO protein has emerged as both a biomarker and a therapeutic target in the inflammatory response. Research indicates that DCR3-SUMO has anti-inflammatory properties. It improves tissue morphology in sepsis models, increases survival rates, and significantly reduces serum levels as well as liver and lung concentrations of various inflammatory markers (115).

#### 4.2.3 Antioxidative stress therapy

Antioxidative stress therapies aim to modulate critical signaling pathways that suppress oxidative stress and inflammation to protect the liver. These pathways include the NF-κB (116), Forkhead box O (FOXO) (117, 118), silent information regulator 1 (SIRT1)/NRF2/heme oxygenase-1(HO-1) pathways (119), and mitogen-activated protein kinases (MAPKs), such as extracellular signal-regulated kinases (ERKs), c-Jun N-terminal kinase (JNKs), and p38 (120). NAC has been demonstrated to ameliorate liver dysfunction by reducing lipid peroxides and increasing antioxidant capacity (121, 122). Endocrine and metabolic regulation play crucial roles in modulating oxidative stress. Melatonin downregulates the expression of TLR4 and NF-κB, inhibits the production of ROS and RNS, and upregulates the antioxidant defense system (123, 124). In addition, it activates the SIRT3 pathway in mitochondria, protecting them from oxidative stress and alleviating liver dysfunction and glucose metabolism disorders (125, 126). Additionally, melatonin decreases the expression of iNOS mRNA in the livers of septic mice, decreasing nitric oxide (NO) production and protecting the liver (127, 128). Metformin further alleviates liver injury in sepsis models by reducing cytokine levels and high mobility group protein B1 (HMGB1) and MAPK activation while increasing adenosine 5’-monophosphate (AMP)-activated protein kinase (AMPK) expression (129).

#### 4.2.4 Inhibition of pyroptosis

The activation of the NLRP3 inflammasome is a critical step in the pyroptotic process, and its upstream activators have been extensively studied. Mitochondrial ROS and lysosomal damage are two major upstream activators of the NLRP3 inflammasome. Mitochondrial dysfunction during sepsis leads to increased production of ROS, which can trigger the NLRP3 inflammasome (130). Additionally, lysosomal rupture and the release of cathepsins can also activate the NLRP3 inflammasome, contributing to pyroptosis (131).

Targeting pyroptosis is a promising therapeutic strategy for mitigating liver injury in sepsis. Several therapeutic inhibitors of pyroptosis are currently under investigation. Small-molecule inhibitors targeting GSDMD have shown potential in preclinical studies (132). For example, the compound Vx-765 has been reported to inhibit GSDMD cleavage and pyroptosis, thereby reducing inflammation and tissue injury (133–135). Moreover, inhibitors targeting the NLRP3 inflammasome itself are also in development (136). The compound MCC950 is a potent NLRP3 inflammasome inhibitor that has demonstrated efficacy in animal models of sepsis (135, 137). Drug-free tea polyphenols nanoparticles (TPNs) have intrinsic broad-spectrum RONS scavenging and pyroptosis blocking activity, and their ability to inhibit pyroptosis has been clearly demonstrated in a mouse model of sepsis and in human cells (138).

These therapeutic inhibitors provide new avenues for the treatment of sepsis-induced liver injury and warrant further investigation in clinical trials.

#### 4.2.5 Chinese medicine treatment

Traditional Chinese medicine (TCM) has already exhibited advantages in the treatment of SILI. The principal TCM active ingredients, curcumin and forskolin, exhibit anti-inflammatory, antioxidant, and hepatoprotective effects (36). Ginsenoside Rg3 alleviates SILI by modulating the lncRNA TUG1/miR-200c-3p/SIRT1 signaling axis (139). Moreover, Baweidu powder (BWBDS) ameliorates SILI through the modulation of the gut microbiota in murine models, emphasizing the therapeutic role of the microbiome in hepatic protection (140). Therefore, there is substantial research potential for the use of TCM in treating SILI.

#### 4.2.6 Regulating autophagy

Targeting autophagy pathways has emerged as a promising approach for alleviating SILI. Specific inhibitors of the pyroptosis pathway, such as those that activate peroxisome proliferator-activated receptor γ (PPARγ), can inhibit the thioredoxin-interacting protein (TXNIP)/NLRP3 signaling pathway (141). Moreover, immune-responsive gene 1 (IRG1)/4-octyl itaconate (OI) regulates the Nrf2 signaling pathway to suppress NLRP3 inflammasome activation and macrophage pyroptosis, reducing acute liver injury in septic mice (142). Furthermore, compounds such as anemonin-B4 and albiflorin increase the expression of autophagy-related proteins via the mTOR/p70S6K signaling pathway (143, 144), whereas dexmedetomidine regulates the SIRT1/AMPK axis (145). Acetaldehyde dehydrogenase-2 (ALDH2) also mediates vital signaling proteins, including AKT, AMPK, mTOR, STAT3, and Notch1, which cooperatively increase the flux of hepatic autophagy and prevent hepatocyte damage (146). These findings provide compelling evidence for the therapeutic potential of autophagy regulation in the treatment of SILI.

#### 4.2.7 Epigenetic treatment

Epigenetic modulation of inflammatory pathways is a promising approach for SILI treatment. Strategies targeting specific miRNAs have been identified to modulate key signaling pathways, such as the NF-κB (87, 147), TLR4 (148), and MAPK (149)pathways. For example, several reports have demonstrated that paclitaxel ameliorates liver injury in septic mice by modulating the miR-27a/TAB3/NF-κB axis (150). The overexpression of miR-103a-3p targets HMGB1, leading to reduced inflammation and cellular damage, thereby improving SILI (151). Moreover, inhibiting miR-155 to promote the expression of suppressor of cytokine signaling (SOCS1) to inhibit the JAK/STAT pathway has been found to decrease SILI (152). The lncRNA CASC9 offers another approach for treating SILI by stabilizing SOCS1 through its interaction with fused in sarcoma (FUS) (153). Additionally, the inhibition of the lncRNA LINC00472 and its regulation of the miR-373-3p/TRIM8 axis has also presented potential benefits in alleviating SILI (154). Silencing the lncRNA XIST reduces BRD4 expression, which evades sepsis-induced acute liver injury, and could serve as a novel biomarker for diagnosing and treating sepsis (155). Although these findings are promising in preclinical models, there is an urgent need for future research to translate these strategies into clinical applications.

#### 4.2.8 Cell therapy

Mesenchymal stem cells (MSCs) have garnered significant attention as promising cell-based therapeutic strategies for the treatment of sepsis owing to their wide availability, high self-renewal capacity, multilineage differentiation potential, and low immunogenicity. Studies conducted in animal models have reported that MSCs can prevent liver inflammation during sepsis (156, 157). Human Wharton’s Jelly-derived mesenchymal stem cells (WJ-MSCs) significantly attenuate the expression of NF-κB and cytokines, thereby alleviating SILI in a murine model of sepsis (158). More specifically, MSCs mitigate SILI by suppressing the M1 polarization of KCs (159). In addition to whole MSCs, exosomes derived from MSCs have emerged as an applicable alternative. Exosomes are capable of encapsulating various bioactive molecules, such as RNA, cytokines, chemokines, proteins, and lipids (160). Through paracrine modulation, these exosomes mediate intracellular and intercellular signaling as well as metabolic processes (161). For example, exosomes alleviate sepsis-associated acute liver injury by downregulating MALAT1 via miRNA-26a-5p (162). Nevertheless, two major limitations affecting the clinical application of MSCs include their low efficiency in differentiation and minimal survival rates post transplantation. However, MSC-derived exosomes provide an alternative opportunity to increase therapeutic specificity and precision in treating sepsis while addressing these challenges.

## 5 Discussion and prospects

Liver injury in sepsis is a complex pathophysiological process. The main factors are inflammatory mediators, oxidative stress, and apoptosis. Although these mechanisms have not been fully elucidated, further studies on the mechanism of SILI may lead to the identification of new therapeutic targets. These discoveries hold promise not only for advancing treatment strategies for septic liver injury but also for enhancing overall management approaches for sepsis.

Otherwise, clinical decision-making can be significantly enriched if further research identifies biomarkers that indicate disease status, predict disease progression, and monitor treatment efficacy. Such biomarkers would prove invaluable in selecting appropriate personalized treatments and optimizing patient outcomes. In parallel, it is essential to develop safe, effective, and accessible treatments that address the diverse needs of patients. The simplification of procedures, reduction in treatment duration, and decreased costs will also further optimize the existing treatment protocols and improve the overall efficacy of interventions for SILI.

## Glossary

AKT: Protein kinase B
ALDH: Acetaldehyde dehydrogenase-2
ALP: Alkaline phosphatase
AMPK: Adenosine 5’-monophosphate (AMP)-activated protein kinase
ARDS: Acute respiratory distress syndrome
BWBDS: Baweidu powder
CLP: Cecal ligation and perforation
DAMPs: Damage-associated molecular patterns
DcR3: Decoy Receptor 3
ERK: Extracellular signal-regulated kinases
FOXO: Forkhead box O
FUS: Fused in sarcoma
GGT: γ-glutamyltransferase
GPX4: Glutathione peroxidase 4
GSDMD: Gasdermin-D
HH: Hypoxic hepatitis
HMGB1: High mobility group protein B1
HO-1: Heme oxygenase-1
ICU: Intensive care unit
IFIH1: Interferon-induced helicase C-domain protein 1
IL-1β: Interleukin-1β
IL-6: Interleukin-6
IL-8: Interleukin-8;INR, International normalized ratio
IRG1: Immune-responsive gene 1
JNK: c-Jun N-terminal kinase
KCs: Kupffer cells
LDH: Lactate dehydrogenase
LPSs: Lipopolysaccharides
MAPKs: Mitogen-activated protein kinases
MODS: Multiple organ dysfunction syndrome
MRCP: Magnetic resonance cholangiopancreatography
MSCs: Mesenchymal stem cells
mTOR: Mechanistic target of rapamycin
NAC: N-acetylcysteine
NF-κB: Nuclear factor-κB
NLRP3: NOD-like receptor protein 3
NO: Nitric oxide
Nrf2: Nuclear factor E2-related factor 2
OI: 4-octyl itaconate
PAMPs: Pathogen-associated molecular patterns
PI3K: Phosphoinositide 3-kinase
PPARγ: Peroxisome proliferator-activated receptor γ
PRRs: Pattern recognition receptors
PTBP1: Polypyrimidine tract-binding protein 1
RNS: Reactive nitrogen species
ROS: Reactive oxygen species
SC: Septic cholestasis
SILI: Sepsis-induced liver injury
SIRS: Systemic inflammatory response syndrome
SIRT1: Silent information regulator 1
SOCS1: Suppressor of cytokine signaling
SSC: Secondary sclerosing cholangitis
TCM: Traditional Chinese medicine
TLRs: Toll-like receptors
TNF-α: Tumor necrosis factor-α
TXNIP: Thioredoxin-interactingprotein
